# A Functional Evaluation of Resveratrol–Paclitaxel Combination Reveals Enhanced Apoptotic Responses in HeLa Cells

**DOI:** 10.3390/ijms27104505

**Published:** 2026-05-18

**Authors:** Elif Ozan, Mehmet Cudi Tuncer, İlhan Özdemir

**Affiliations:** 1 Department of Gynecology and Obstetrics, Dr. Elif Ozan Practice, 06690 Ankara, Turkey; drelifozan@hotmail.com; 2 Department of Anatomy, Faculty of Medicine, Dicle University, 21280 Diyarbakır, Turkey; 3 Department of Histology Embryology, Faculty of Medicine, Kahramanmaraş Sütçü İmam University, 46000 Kahramanmaraş, Turkey; ilhanozdemir32@hotmail.com

**Keywords:** apoptosis, HeLa cells, caspase activation, mitochondrial dysfunction, resveratrol, cervical cancer

## Abstract

This study evaluated the combined effects of resveratrol (RES) and paclitaxel (PAC) on cell viability, apoptotic responses, and associated cellular processes in HeLa cervical cancer cells. Antiproliferative activity was assessed using XTT assay and combination index (CI) analysis, while apoptosis, cell cycle distribution, mitochondrial membrane potential (ΔΨm), and intracellular reactive oxygen species (ROS) levels were examined by flow cytometry-based approaches. The RES + PAC combination produced a synergistic reduction in cell viability compared to single treatments. This effect was accompanied by increased apoptotic cell populations and a marked accumulation of cells in the G2/M phase. Combined treatment was also associated with a pronounced loss of mitochondrial membrane potential and elevated ROS levels. Gene expression analysis indicated an increased *Bax/Bcl-2* mRNA ratio together with upregulation of apoptosis-related markers and downregulation of cell cycle regulators. Importantly, pharmacological inhibition of ROS using N-acetylcysteine (NAC) partially attenuated both ROS accumulation and the reduction in cell viability, suggesting that oxidative stress contributes, but is not solely responsible, for the observed cytotoxic effects. Overall, these findings indicate that the combination of RES and PAC enhances apoptotic responses in HeLa cells through mechanisms associated with mitochondrial dysfunction, oxidative stress, and cell cycle perturbation. Further studies are required to clarify the underlying pathways and to evaluate the translational relevance of these findings.

## 1. Introduction

Cervical cancer remains a major global health burden, with high incidence and mortality rates particularly in low- and middle-income countries [1]. Persistent infection with high-risk human papillomavirus (HPV), especially types 16 and 18, is recognized as the primary etiological factor responsible for the majority of cases [2]. Chemotherapeutic agents such as PAC, carboplatin, and cisplatin are widely used in advanced disease; however, overall survival remains limited [3]. PAC is a naturally occurring diterpenoid compound originally isolated from the bark of Taxus brevifolia and is widely used as a first-line chemotherapeutic agent in the treatment of multiple solid tumors [4]. PAC exerts its cytotoxic effect primarily through stabilization of microtubules by binding to β-tubulin, leading to cell cycle arrest in the G2/M phase [5]. Prolonged mitotic arrest subsequently activates apoptotic signaling pathways [6], including mechanisms associated with modulation of the *Bax/Bcl-2* balance [7]. Despite its clinical utility, the effectiveness of PAC is often reduced due to the development of resistance mechanisms, including alterations in tubulin dynamics and apoptotic signaling pathways [8].

Naturally derived compounds have increasingly been explored as adjuvant strategies to improve chemotherapy outcomes. Among these, polyphenols have been reported to enhance chemosensitivity and modulate drug resistance mechanisms [9]. Several studies have demonstrated that natural compounds, including RES, can exert synergistic antitumor effects in combination with chemotherapeutic agents such as cisplatin, doxorubicin, PAC, and 5-fluorouracil [10,11,12]. RES, a polyphenolic compound found in various plant sources, has been shown to exert antiproliferative, pro-apoptotic, and redox-modulating effects in different cancer cell types [13]. In cervical cancer models, RES has been reported to regulate *Bax/Bcl-2* balance, activate caspase signaling, and alter mitochondrial function, thereby influencing cell survival pathways [14,15,16].

Natural compounds have increasingly attracted attention as adjuvant agents in cancer therapy due to their potential to enhance treatment efficacy while mitigating toxicity. Among these, RES, a naturally occurring polyphenolic compound, has been widely investigated for its antioxidant, anti-inflammatory, and anticancer properties. Recent evidence suggests that RES may modulate tumor resistance mechanisms and improve the therapeutic index of conventional anticancer treatments, including chemotherapy and radiotherapy [17]. In this context, RES has been proposed as a promising adjunct capable of enhancing the efficacy of established chemotherapeutic agents while potentially reducing adverse effects on normal tissues.

Several recent studies have explored the combinatorial use of RES with chemotherapeutic agents such as PAC, reporting enhanced cytotoxic and pro-apoptotic effects in different cancer models. For example, RES has been shown to potentiate PAC-induced apoptosis through mechanisms associated with increased ROS production, oxidative DNA damage, and modulation of antioxidant defense systems [18]. In addition, emerging formulation-based approaches, such as co-amorphous drug systems, have demonstrated that the combination of RES with PAC can improve drug solubility, bioavailability, and overall antitumor efficacy, further supporting the potential of this combination strategy [19].

However, despite these promising findings, the biological effects of RES remain complex and, in some contexts, controversial. Evidence indicates that RES does not act through a single dominant molecular target but instead interacts with multiple signaling pathways, resulting in heterogeneous and sometimes inconsistent biological outcomes across experimental systems [20]. In this context, RES has been reported to exert a wide range of biological effects, including modulation of oxidative stress, apoptosis, and tumor-related signaling processes, although these effects may vary depending on cellular conditions, treatment duration, and concentration. Notably, RES has been described as a compound capable of exhibiting both antioxidant and pro-oxidant activities, as well as dual effects on cell survival and death pathways, further highlighting its pleiotropic nature [21]. Moreover, despite extensive preclinical and clinical investigation, no clear consensus has been reached regarding the precise mechanisms underlying RES’s biological activity or its therapeutic applicability in cancer treatment [20].

In line with this complexity, accumulating evidence indicates that RES may exert context-dependent effects, including the potential to attenuate the cytotoxic activity of chemotherapeutic agents under specific molecular conditions. For instance, RES has been reported to reduce PAC-induced cytotoxicity in certain breast cancer cell lines through activation of the SIRT1–FOXO1–HER3 signaling axis, thereby promoting drug resistance [22]. These findings highlight that the interaction between RES and PAC is not uniformly synergistic and may involve both enhancing and inhibitory effects depending on cellular context. Therefore, further experimental studies are required to clarify the conditions under which RES acts as a beneficial adjuvant and to better define the underlying cellular responses associated with RES–PAC combination treatment.

Despite these promising findings, the interaction between RES and PAC remains complex and context-dependent. While synergistic cytotoxic effects have been reported [17,23], other studies suggest that the antioxidant properties of RES may attenuate PAC-induced oxidative stress, potentially reducing its efficacy under certain conditions [18,24]. This apparent discrepancy indicates that the biological outcome of this combination may depend on cellular context and experimental conditions, highlighting the need for a more detailed mechanistic evaluation.

Based on these considerations, the present study aimed to investigate the effects of RES and PAC, alone and in combination, on HeLa cervical cancer cells, with a particular focus on apoptosis, cell cycle regulation, mitochondrial membrane potential, and ROS-associated cellular responses. In addition, the contribution of oxidative stress to the observed effects was further examined using pharmacological modulation.

## 2. Results

### 2.1. Synergistic Antiproliferative Effects of Res and Pac Revealed by Dose–Response and Ci Analyses

**Figure 1:**
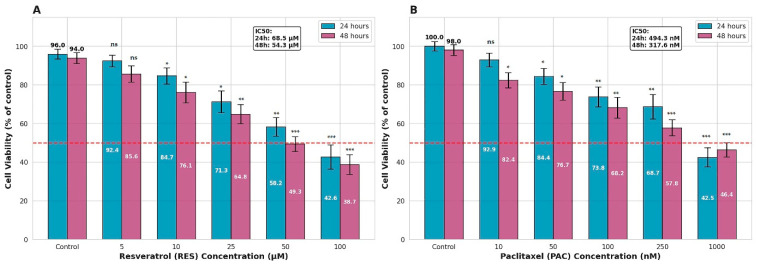
Dose- and time-dependent antiproliferative effects of RES and PAC in HeLa cells. HeLa cervical cancer cells were treated with increasing concentrations of RES (5–100 µM) (**A**) or PAC (10–1000 nM) (**B**) for 24 and 48 h. Cell viability was assessed using the XTT assay and expressed as percentage relative to untreated control cells. Data are presented as mean ± SD of three independent experiments (*n* = 3). The dashed red line indicates the 50% viability threshold. Both RES and PAC exhibited dose- and time-dependent reductions in cell viability, with lower IC_50_ values observed at 48 h compared to 24 h, indicating increased antiproliferative potency over time. IC_50_ values were calculated by log-linear interpolation and are shown within each panel (RES: 68.5 µM at 24 h and 54.3 µM at 48 h; PAC: 494.3 nM at 24 h and 317.6 nM at 48 h). Statistical significance was determined by one-way ANOVA followed by Tukey’s post hoc test. * *p* < 0.05, ** *p* < 0.01, *** *p* < 0.001 versus control. ns: not significant. For RES, statistically significant reductions in cell viability were observed at concentrations ≥10 µM at both 24 h (*p* = 0.031, 0.018, 0.0065, and 0.0004 for 10–100 µM, respectively) and 48 h (*p* = 0.028, 0.014, 0.0049, and 0.0003 for 10–100 µM, respectively), while 5 µM remained non-significant at both time points (*p* = 0.082 and 0.061). For PAC, significant effects were detected at concentrations ≥ 10 nM at both 24 h (*p* = 0.029, 0.021, 0.0082, 0.0058, and 0.0005 for 10–1000 nM, respectively) and 48 h (*p* = 0.027, 0.018, 0.0076, 0.0042, and 0.0004 for 10–1000 nM, respectively).

RES and PAC exhibited dose- and time-dependent antiproliferative effects on HeLa cervical carcinoma cells when applied individually and in combination. At 24 h, RES treatment resulted in cell viability values of 92.4 ± 3.1%, 84.7 ± 4.2%, 71.3 ± 5.6%, 58.2 ± 4.8%, and 42.6 ± 6.3% at concentrations of 5, 10, 25, 50, and 100 µM, respectively, corresponding to an half-maximal inhibitory concentration (IC_50_) value of 68.5 µM (Figure 1A). Under the same conditions, PAC treatment yielded viability rates of 92.9 ± 3.5%, 84.4 ± 4.1%, 73.8 ± 5.2%, 68.7 ± 6.4%, and 42.5 ± 4.9% at concentrations of 10, 50, 100, 250, and 1000 nM, respectively, with an IC_50_ value of 494.3 nM.

**Figure 2:**
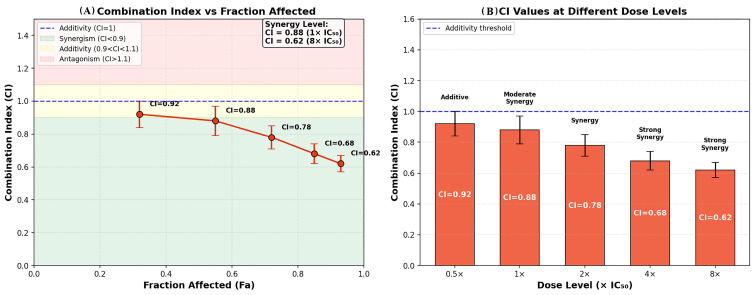
Quantitative analysis of the synergistic interaction between RES and PAC using the Chou–Talalay method. (**A**) CI values plotted against fraction affected (Fa), demonstrating the relationship between drug effect and interaction type. CI values below 1 indicate synergism, with decreasing CI values at higher Fa levels reflecting increased synergy at greater levels of growth inhibition. (**B**) CI values at different dose levels expressed as multiples of the IC_50_ (0.5×, 1×, 2×, 4×, and 8× IC_50_). The combination exhibited a progressive decrease in CI values from 0.92 to 0.62, indicating a shift from near-additive effects at lower doses to moderate and strong synergism at higher dose levels. Data are presented as mean ± SD of three independent experiments (*n* = 3). The dashed line represents the additivity threshold (CI = 1). CI values were calculated from triplicate experiments and are presented as mean ± SD. Dose levels were defined based on IC_50_ values obtained from 48 h dose–response analyses. CI values were interpreted as follows: CI < 0.9, synergism; 0.9–1.1, additive effect; CI > 1.1, antagonism. Statistical analysis was performed using one-way ANOVA followed by Tukey’s post hoc test, with *p* < 0.05 considered statistically significant.

In the combination setting, RES and PAC were applied at a fixed concentration ratio selected within IC_50_-based exposure ranges. The selected combination doses represent approximate IC_50_-based exposure ranges rather than a strict equipotent ratio design. The combined treatment (100 µM RES + 1000 nM PAC) reduced cell viability to 37.2 ± 5.7% (Figure 1B), indicating a stronger inhibitory effect compared to single-agent treatments. Quantitative assessment using the Chou–Talalay method revealed a CI value of 0.88, corresponding to moderate synergism. Consistent with this finding, the combination treatment produced a significantly greater reduction in cell viability compared to individual treatments (*p* < 0.01) (Figure 2).

Following 48 h treatment, both RES and PAC exhibited enhanced antiproliferative effects compared to 24 h exposure. RES treatment resulted in cell viability values of 85.6 ± 4.2%, 76.1 ± 5.3%, 64.8 ± 4.9%, 49.3 ± 3.8%, and 38.7 ± 5.1% at concentrations of 5, 10, 25, 50, and 100 µM, respectively, corresponding to an IC_50_ value of 54.3 µM (Figure 1A). Similarly, PAC treatment yielded viability rates of 82.4 ± 3.9%, 76.7 ± 4.6%, 68.2 ± 5.4%, 57.8 ± 4.2%, and 46.4 ± 3.7% at concentrations of 10, 50, 100, 250, and 1000 nM, respectively, with an IC_50_ value of 317.6 nM (Figure 1B). These findings indicate a time-dependent increase in antiproliferative potency for both agents.

In the combination setting, treatment with RES (100 µM) and PAC (1000 nM) further reduced cell viability to 32.4 ± 4.6%, demonstrating a stronger inhibitory effect than either agent alone. CI analysis revealed a dose-dependent decrease in CI values, ranging from 0.92 ± 0.015 at 0.5× IC_50_ to 0.62 ± 0.020 at 8× IC_50_, indicating progressively enhanced synergistic interaction with increasing exposure (CI < 1). Consistent with this observation, dose reduction index (DRI) analysis suggested that the effective doses of PAC and RES could be reduced by approximately 2.8-fold and 3.1-fold, respectively, under combination conditions (Figure 2).

**Figure 3:**
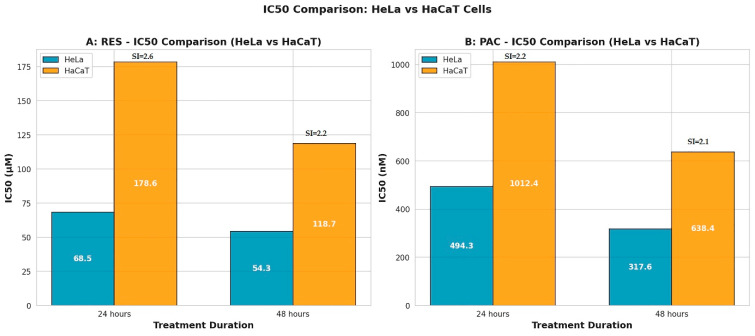
Limited selectivity in immortalized normal cells of RES and PAC in HeLa and HaCaT cells. (**A**) IC_50_ values for RES in HeLa cervical cancer cells and HaCaT normal keratinocytes at 24 and 48 h. (**B**) IC_50_ values for PAC under the same conditions. For both agents, IC_50_ values were consistently higher in HaCaT cells compared to HeLa cells at both time points, indicating reduced sensitivity of normal cells. The selectivity index (SI), calculated as IC_50_(HaCaT)/IC_50_(HeLa), exceeded 2 in all conditions (RES: 2.6 and 2.2; PAC: 2.2 and 2.1 at 24 and 48 h, respectively), indicating limited selectivity in immortalized normal cells rather than definitive tumor-specific selectivity. Data are derived from three independent experiments (*n* = 3). IC_50_ values were calculated by nonlinear regression analysis of dose–response curves. It should be noted that HaCaT cells are immortalized keratinocytes and do not fully represent primary normal cervical epithelial cells.

To assess selectivity, the effects of RES and PAC were also evaluated in normal human keratinocyte (HaCaT) cells. The IC_50_ values in HaCaT cells were consistently higher than those observed in HeLa cells at both time points. At 24 h, IC_50_ values were 178.6 µM for RES (SI = 2.6) and 1012.4 nM for PAC (SI = 2.2). At 48 h, IC_50_ values were 118.7 µM (SI = 2.2) for RES and 638.4 nM (SI = 2.1) for PAC (Figure 3A,B), indicating preferential cytotoxicity toward cancer cells.

### 2.2. Enhanced Apoptotic Induction by Combined Res and Pac Treatment

**Figure 4:**
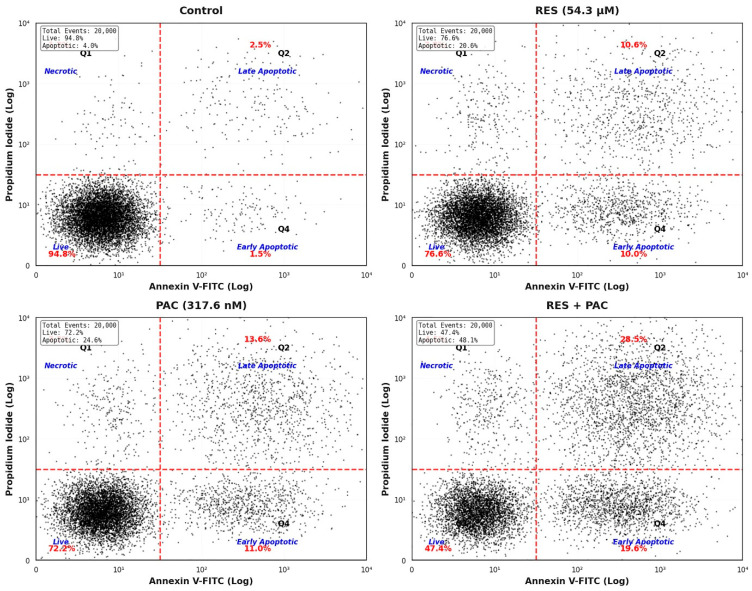
Flow cytometric analysis of apoptosis induced by RES, PAC, and their combination in HeLa cells. Representative Annexin V-FITC/PI dot plots of HeLa cells following 48 h treatment with RES, PAC, and their combination at IC_50_ concentrations. Cells were categorized as viable (Annexin V^−^/PI^−^), early apoptotic (Annexin V^+^/PI^−^), late apoptotic (Annexin V^+^/PI^+^), and necrotic (Annexin V^−^/PI^+^). RES treatment increased the total apoptotic cell population compared to control cells, with elevations in both early and late apoptotic fractions. PAC treatment resulted in a higher proportion of apoptotic cells than RES, affecting both early and late apoptotic populations. The combination of RES and PAC produced the highest level of apoptosis among all groups, with a pronounced accumulation of both early and late apoptotic cells, suggesting an enhanced apoptotic response under combination conditions. Data are representative of flow cytometry experiments and are presented for illustrative purposes. Annexin V/PI staining does not allow definitive discrimination between late apoptosis and secondary necrosis.

The apoptotic effects of RES and PAC, alone and in combination, were evaluated in HeLa cells using Annexin V-FITC/propidium iodide (PI) double staining followed by flow cytometry. Analyses were performed after 48 h of treatment at IC_50_ concentrations. Representative dot plots illustrating apoptotic cell distributions are shown in Figure 4.

In the control group (0.1% DMSO), the total apoptotic cell population (early + late apoptosis) was 5.2 ± 1.1%. Treatment with RES increased total apoptosis to 23.4 ± 3.2%, with contributions from early apoptotic cells (12.8 ± 2.1%) and late apoptotic/necrotic cells (10.6 ± 1.8%). Similarly, PAC treatment resulted in a total apoptotic fraction of 27.8 ± 4.1%, comprising 14.2 ± 2.4% early apoptotic and 13.6 ± 2.3% late apoptotic/necrotic cells.

Combined treatment with RES and PAC markedly increased the total apoptotic population to 52.6 ± 5.7%, representing a substantial elevation compared to single-agent treatments (*p* < 0.001). This increase was observed in both early and late apoptotic fractions, indicating an overall enhancement of apoptotic cell death under combination conditions (Figure 4).

### 2.3. Res and Pac Combination Enhances G2/M Arrest and Sub-G1 Accumulation in Hela Cells

**Figure 5:**
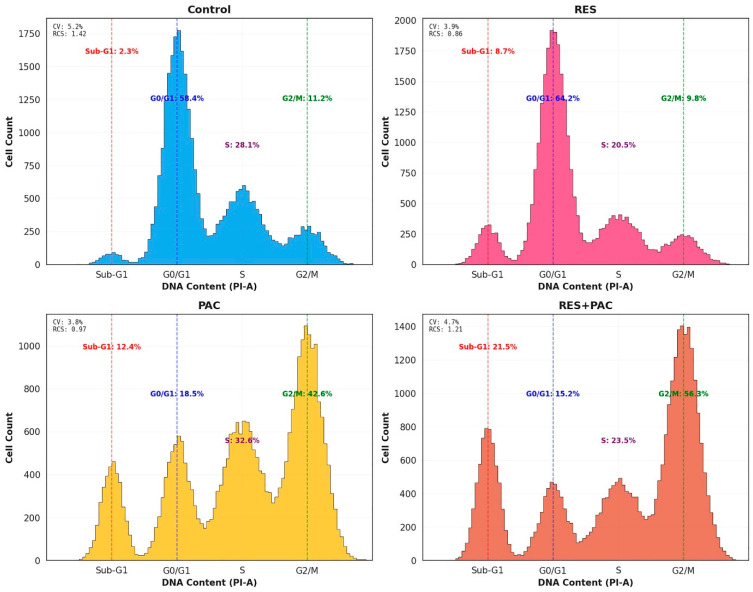
Cell cycle distribution of HeLa cells following treatment with RES, PAC, and their combination. Representative DNA content histograms obtained by PI staining and flow cytometry analysis after 48 h treatment with RES (54.3 µM), PAC (317.6 nM), and their combination at IC_50_ concentrations. Cell populations were distributed across sub-G1, G0/G1, S, and G2/M phases. Control cells exhibited a typical distribution with the majority of cells in the G0/G1 phase. RES treatment resulted in a modest increase in the G0/G1 population and an elevation in the sub-G1 fraction, often associated with apoptotic DNA fragmentation. PAC treatment led to a pronounced accumulation of cells in the G2/M phase, accompanied by an increase in the sub-G1 population. The combination of RES and PAC further enhanced these effects, with a marked increase in G2/M phase accumulation and a substantial rise in the sub-G1 fraction compared to single-agent treatments, suggesting an association with increased G2/M accumulation and apoptotic cell enrichment. Data are representative of flow cytometry experiments and are presented for illustrative purposes. Cell cycle distribution was determined using PI staining and DNA content analysis.

Cell cycle distribution in HeLa cells was analyzed following treatment with RES, PAC, and their combination. In control cells, the distribution was 58.4 ± 2.3% in G0/G1, 28.1 ± 1.9% in S phase, 11.2 ± 1.5% in G2/M, and 2.3 ± 0.8% in the sub-G1 fraction (Figure 5).

Treatment with RES resulted in a modest increase in the G0/G1 population (64.2%) accompanied by an elevation in the sub-G1 fraction (8.7%), indicating limited cell cycle perturbation with increased apoptotic cell accumulation. In contrast, PAC treatment led to a pronounced accumulation of cells in the G2/M phase (42.6 ± 3.8%), along with an increase in the sub-G1 population (12.4%).

Combined treatment with RES and PAC further enhanced these effects, with the G2/M fraction increasing to 56.3 ± 4.2% and the sub-G1 population rising to 21.5 ± 2.9%. This combined effect was significantly greater than that observed with single-agent treatments (*p* < 0.01), indicating enhanced cell cycle arrest and apoptotic cell accumulation under combination conditions (Figure 5).

### 2.4. Res and Pac Combination Promotes Mitochondrial Membrane Depolarization in Hela Cells

**Figure 6:**
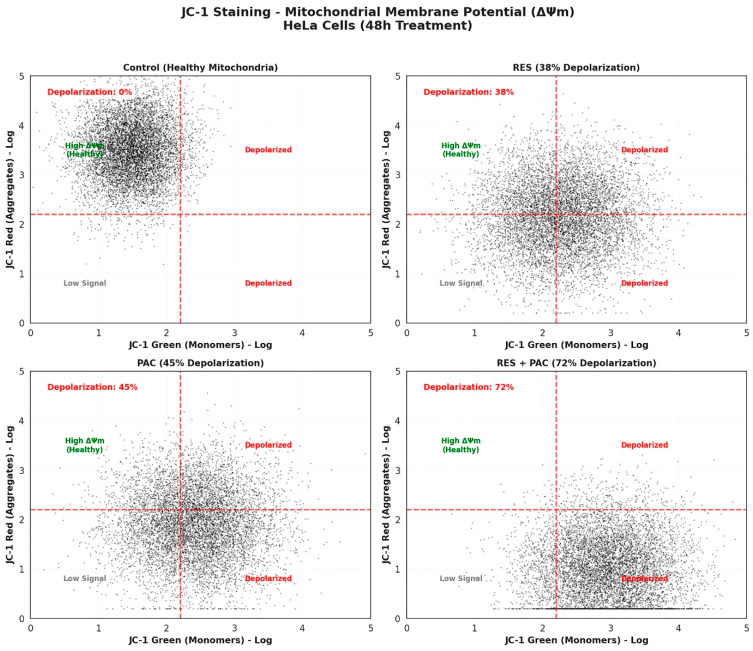
JC-1 analysis of mitochondrial membrane potential (ΔΨm) in HeLa cells following treatment with RES, PAC, and their combination. Representative JC-1 dot plots illustrate mitochondrial membrane potential after 48 h treatment with RES, PAC, and their combination at IC_50_ concentrations. The distribution of cells reflects mitochondrial polarization status, where high red fluorescence (JC-1 aggregates) indicates preserved ΔΨm, whereas increased green fluorescence (JC-1 monomers) is associated with mitochondrial depolarization. Control cells predominantly exhibited high red fluorescence, consistent with intact mitochondrial membrane potential. Treatment with RES and PAC individually was associated with an increased proportion of depolarized cells. The percentage of depolarized cells was determined based on quadrant distribution and is indicated in each panel: RES (38%), PAC (45%), and RES + PAC (72%). The combined treatment (RES + PAC) further increased the proportion of depolarized cells, suggesting an enhanced loss of ΔΨm compared to single-agent treatments.

Mitochondrial membrane potential (ΔΨm) was evaluated using JC-1 staining followed by flow cytometry analysis after 48 h treatment with RES, PAC, and their combination (Figure 6). In control cells, a high red/green fluorescence ratio (JC-1 aggregates/monomers) was observed, indicating intact mitochondrial membrane potential. Treatment with RES and PAC individually resulted in reductions in the red/green fluorescence ratio by 38% and 45%, respectively, indicating partial mitochondrial depolarization. In contrast, combined treatment with RES and PAC led to a more pronounced decrease in the red/green ratio (72 ± 6.4%), consistent with a substantial loss of mitochondrial membrane potential.

### 2.5. Res and Pac Combination Enhances Intracellular Ros Accumulation in Hela Cells

**Figure 7:**
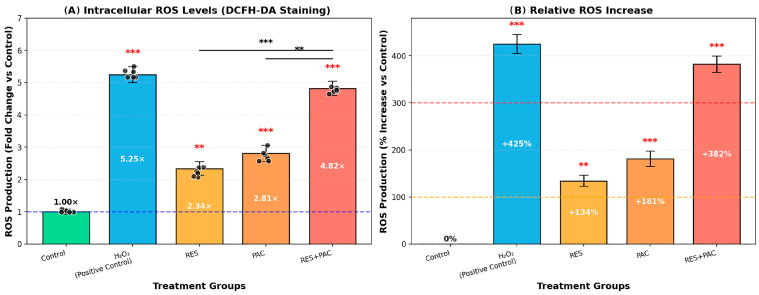
Intracellular ROS levels in HeLa cells following treatment with RES, PAC, and their combination. Intracellular ROS production was measured using DCFH-DA staining and flow cytometry after 48 h treatment with RES (54.3 µM), PAC (317.6 nM), and their combination. H_2_O_2_ (200 µM) was used as a positive control. A total of 10,000 events were acquired per sample. Autofluorescence was subtracted, and ROS levels were normalized to control values (fold change = MFI(sample)/MFI(control)). (**A**) ROS fold change relative to control: Control (1.00×), H_2_O_2_ (5.45×, *** *p* < 0.001), RES (2.34×, ** *p* < 0.01), PAC (2.81×, *** *p* < 0.001), and RES + PAC (4.82×, *** *p* < 0.001). (**B**) Percentage increase in ROS levels: H_2_O_2_ (+445%), RES (+134%), PAC (+181%), and RES + PAC (+382%). Data are presented as mean ± SD from three independent experiments (*n* = 3). Treatment with RES and PAC individually was associated with increased intracellular ROS levels compared to control. The combined treatment (RES + PAC) further increased ROS levels relative to single-agent treatments; however, this increase did not exceed the levels observed with the H_2_O_2_ positive control. These findings suggest that ROS generation may contribute to the observed cellular responses, although a direct causal relationship cannot be established based on this assay alone.

Intracellular ROS levels were assessed in HeLa cells following treatment with RES, PAC, and their combination using DCFH-DA staining and flow cytometry (Figure 7). Analyses were performed after 48 h treatment at IC_50_ concentrations.

Compared to control cells (normalized to 1.0), RES treatment increased ROS levels by 2.34-fold, while PAC treatment resulted in a 2.81-fold increase. Notably, combined treatment with RES and PAC led to a more pronounced elevation in ROS levels, reaching a 4.82-fold increase (*p* < 0.001).

The increase in ROS observed under combination conditions was significantly greater than that induced by either agent alone (*p* < 0.01), indicating an enhanced oxidative response in HeLa cells (Figure 7).

### 2.6. Nac Partially Attenuates Ros Accumulation and Cytotoxicity Induced by Res and Pac

**Figure 8:**
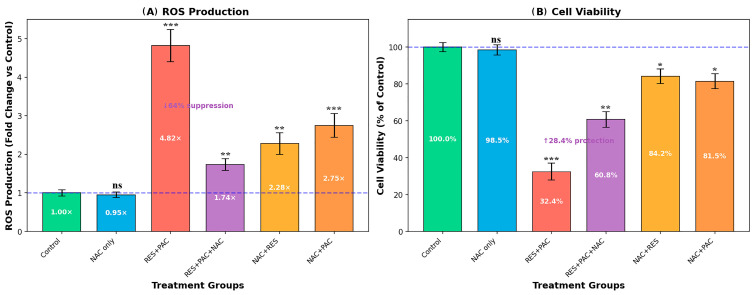
Effects of NAC pre-treatment on ROS levels and cell viability in RES + PAC-treated HeLa cells. HeLa cells were pre-treated with 5 mM NAC for 1 h prior to treatment with RES + PAC for 48 h. (**A**) Intracellular ROS levels expressed as fold change relative to control: Control (1.00×), RES + PAC (4.82×), and RES + PAC + NAC (1.74×). (**B**) Cell viability expressed as percentage of control: Control (100%), RES + PAC (32.4%), and RES + PAC + NAC (60.8%). Data are presented as mean ± SD from three independent experiments (*n* = 3). Statistical significance was determined by one-way analysis of variance (ANOVA) followed by Tukey’s post hoc test (* *p* < 0.05, ** *p* < 0.01, *** *p* < 0.001 vs. control; ns: not significant). NAC pre-treatment partially attenuated both ROS accumulation and the reduction in cell viability observed in the RES + PAC group. NAC pre-treatment was associated with a reduction in intracellular ROS levels and a partial recovery in cell viability in RES + PAC-treated cells. These findings suggest a potential involvement of ROS in the observed cytotoxic effects; however, a direct causal relationship cannot be established based on these data alone.

To investigate the role of oxidative stress, HeLa cells were pre-treated with 5 mM NAC prior to RES, PAC, and combination treatments (Figure 8).

As shown in Figure 8A, the RES + PAC combination markedly increased intracellular ROS levels (4.82-fold vs. control, *p* < 0.001). NAC pre-treatment significantly reduced ROS levels in the RES + PAC + NAC group (1.74-fold, *p* < 0.001 vs. RES + PAC). NAC alone did not significantly affect basal ROS levels (0.95-fold, *p* > 0.05 vs. control).

Similarly, NAC pre-treatment reduced ROS levels in NAC + RES (2.28-fold) and NAC + PAC (2.75-fold) groups compared to RES (2.34-fold) and PAC (2.81-fold) treatments alone, although the magnitude of reduction was less pronounced than that observed in the combination group.

Consistent with these findings, the RES + PAC combination reduced cell viability to 32.4% (*p* < 0.001 vs. control), whereas NAC pre-treatment significantly increased viability to 60.8% in the RES + PAC + NAC group (*p* < 0.01 vs. RES + PAC) (Figure 8B). NAC alone did not significantly affect cell viability (98.5%, *p* > 0.05 vs. control).

In addition, NAC pre-treatment partially restored cell viability in NAC + RES (84.2%) and NAC + PAC (81.5%) groups compared to RES (42.6%) and PAC (42.5%) alone.

### 2.7. Res and Pac Combination Modulates Apoptosis- and Cell Cycle-Related Gene Expression in Hela Cells

**Figure 9:**
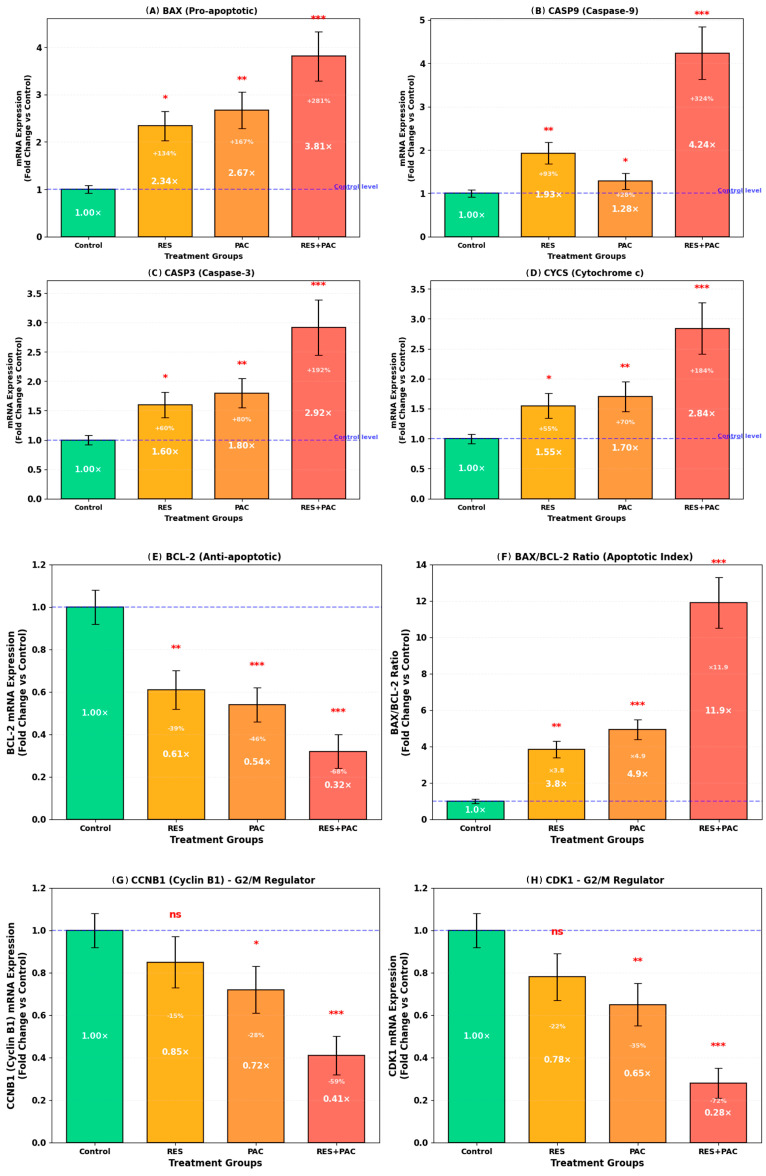
RT-qPCR analysis of apoptosis- and cell cycle-related gene expression in HeLa cells following treatment with RES, PAC, and their combination. HeLa cells were treated with IC_50_ concentrations of RES (54.3 µM), PAC (317.6 nM), and their combination for 48 h. mRNA expression levels were normalized to GAPDH and expressed as fold change relative to control (set to 1.0). Data are presented as mean ± SD from three independent experiments (*n* = 3). (**A**–**D**) Pro-apoptotic genes (*BAX*, *CASP9*, *CASP3*, and *CYCS*) were upregulated following treatment, with the highest fold changes observed in the combination group. Among these, *CASP9* showed the highest fold change (4.24-fold, *** *p* < 0.001). (**E**) The anti-apoptotic gene BCL-2 was downregulated across all treatment groups, with the lowest expression observed in the combination group (0.32-fold, *** *p* < 0.001). (**F**) The *BAX/BCL-2* ratio was increased in the combination group compared to control (*** *p* < 0.001), suggesting a shift toward a pro-apoptotic gene expression profile. (**G**,**H**) Cell cycle regulator genes *CCNB1 (Cyclin B1)* and *CDK1* were downregulated following treatment, with the strongest reductions observed in the combination group (0.41-fold and 0.28-fold, respectively; *** *p* < 0.001), which may be associated with the G2/M phase accumulation observed in cell cycle analysis. Statistical significance was determined by one-way analysis of variance (ANOVA) followed by Tukey’s post hoc test (* *p* < 0.05, ** *p* < 0.01, *** *p* < 0.001 vs. control; ns: not significant).

To further characterize the molecular effects of RES and PAC, mRNA expression levels of key genes associated with apoptosis and cell cycle regulation were analyzed by RT-qPCR (Figure 9).

Among pro-apoptotic markers, *BAX* expression was increased following treatment with RES (2.34 ± 0.31-fold) and PAC (2.67 ± 0.38-fold), with a more pronounced upregulation observed in the combination group (3.81 ± 0.52-fold, *p* < 0.001 vs. control). Similarly, *CASP9* exhibited the highest level of induction, reaching 4.24 ± 0.61-fold in the combination group. *CASP3* and *CYCS* expression levels were also elevated, with fold increases of 2.92 ± 0.47 and 2.84 ± 0.43, respectively, under combination conditions.

In contrast, the anti-apoptotic gene BCL-2 was downregulated following treatment, with the most substantial decrease observed in the combination group (0.32 ± 0.08-fold), compared to RES (0.61-fold) and PAC (0.54-fold) treatments. Consistent with these changes, the *BAX/BCL-2* mRNA ratio was markedly increased in the combination group, indicating a shift toward a pro-apoptotic expression profile.

Regarding cell cycle regulation, expression levels of *CCNB1* and *CDK1* were reduced following treatment, with the greatest decreases observed in the combination group (0.41 ± 0.09-fold and 0.28 ± 0.07-fold, respectively). These changes are consistent with the observed accumulation of cells in the G2/M phase.

### 2.8. Res and Pac Disrupt Microtubule Organization in Hela Cells

**Figure 10:**
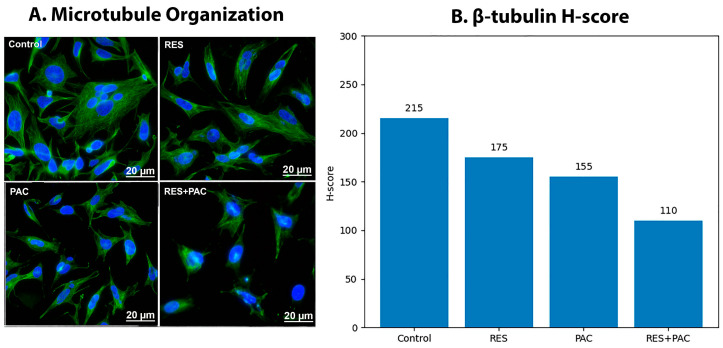
β-tubulin immunofluorescence analysis of microtubule organization in HeLa cells following treatment with RES, PAC, and their combination. (**A**) Representative immunofluorescence images of HeLa cells treated with RES, PAC, and RES + PAC for 48 h. β-tubulin (green) staining illustrates microtubule organization, while nuclei are counterstained with DAPI (blue). Control cells exhibit a dense and well-organized microtubule network. Treatment with RES and PAC was associated with alterations in microtubule organization and a reduction in β-tubulin staining intensity. The combination group showed more pronounced alterations in microtubule organization compared to single-agent treatments. Scale bar: 20 µm. (**B**) Semi-quantitative H-score analysis of β-tubulin staining intensity. A decrease in H-score was observed across treatment groups, with the lowest values detected in the RES + PAC group.

β-tubulin immunofluorescence staining was performed to evaluate microtubule organization in HeLa cells following treatment with RES, PAC, and their combination (Figure 10).

In control cells, a well-organized and dense microtubule network was observed throughout the cytoplasm. Treatment with RES resulted in mild alterations in microtubule structure, accompanied by a reduction in β-tubulin signal intensity. PAC treatment induced more pronounced disruption of microtubule organization, with evident fragmentation of filament structures and changes in cell morphology.

The combination of RES and PAC led to the most substantial alterations, characterized by marked disorganization of the microtubule network and a pronounced decrease in β-tubulin staining intensity. These observations were supported by semi-quantitative H-score analysis, which showed a progressive reduction in β-tubulin signal from control to combination-treated cells.

### 2.9. Res and Pac Combination Increases Caspase-9 Protein Expression in Hela Cells

**Figure 11:**
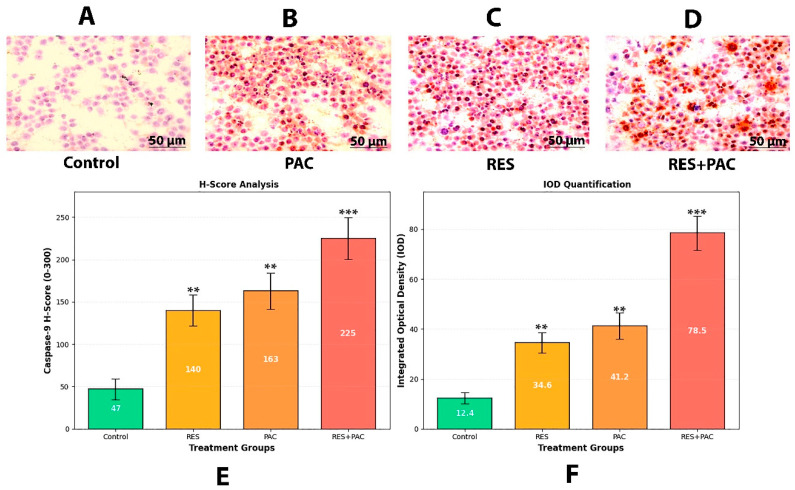
Caspase-9 protein expression in HeLa cells following treatment with RES, PAC, and their combination. (**A**–**D**) Representative immunohistochemical images of HeLa cells treated with control (**A**), RES (**B**), PAC (**C**), and RES + PAC (**D**) for 48 h. Caspase-9 expression is visualized as brown DAB staining, while nuclei are counterstained with hematoxylin (blue). Images were captured at 400× magnification (scale bar: 50 µm). (**E**) Semi-quantitative H-score analysis of caspase-9 expression. (**F**) IOD quantification of caspase-9 immunoreactivity. Both H-score and IOD analyses demonstrate a progressive increase in caspase-9 expression from control to RES- and PAC-treated cells, with the highest levels observed in the RES + PAC combination group. Both H-score and IOD analyses demonstrated increased caspase-9 expression across treatment groups compared to control, with the highest levels observed in the RES + PAC combination group. Data are presented as mean ± SD from three independent experiments (*n* = 3; ≥100 cells analyzed per group). Statistical significance was defined as ** *p* < 0.01 and *** *p* < 0.001 vs. control.

Immunocytochemical staining was performed to evaluate caspase-9 protein expression in HeLa cells following treatment with RES, PAC, and their combination (Figure 11). Caspase-9 immunoreactivity was visualized as brown cytoplasmic staining.

Control cells exhibited minimal caspase-9 staining, consistent with low basal expression levels, with an integrated optical density (IOD) value of 12.4 ± 2.3. Treatment with RES increased caspase-9 expression, as reflected by enhanced cytoplasmic staining and a higher IOD value (34.6 ± 4.1, *p* < 0.01 vs. control).

PAC treatment further elevated caspase-9 expression (41.2 ± 5.3, *p* < 0.01 vs. control), indicating a stronger effect compared to RES alone.

The combination of RES and PAC resulted in the highest level of caspase-9 expression among all groups, with markedly increased cytoplasmic staining intensity and an IOD value of 78.5 ± 6.8 (*p* < 0.001 vs. control). In addition, caspase-9 expression in the combination group was significantly higher than that observed with RES (*p* < 0.001) or PAC (*p* < 0.01) alone.

These findings are consistent with enhanced activation of apoptosis-associated signaling under combination treatment conditions.

### 2.10. Gene Ontology Analysis Indicates Enrichment of Apoptosis- and ROS-Related Pathways

**Figure 12:**
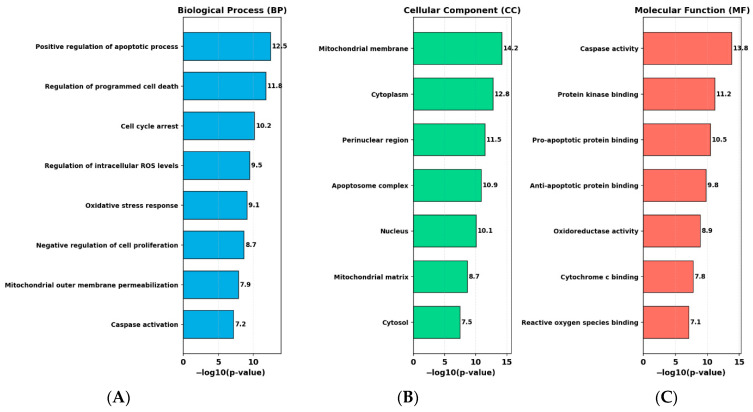
GO enrichment analysis of RES and PAC-associated target genes. (**A**–**C**) Top enriched GO terms categorized into Biological Process (BP), Cellular Component (CC), and Molecular Function (MF). Enrichment significance is presented as −log10(*p*-value), with higher values indicating stronger enrichment (*p* < 0.05, FDR < 0.05). BP analysis identified enrichment of terms associated with apoptotic processes, cell cycle regulation, and oxidative stress-related pathways. CC analysis indicated enrichment in mitochondrial and intracellular components, including mitochondrial membrane and apoptosome-related structures. MF analysis revealed enrichment in functions such as caspase activity, protein binding interactions, and oxidoreductase-related activities. These enrichment patterns suggest potential associations with apoptosis-related processes, ROS-associated signaling, and mitochondrial function; however, these findings are based on in silico analysis and should be interpreted as hypothesis-generating rather than mechanistic evidence.

GO enrichment analysis was performed using DAVID v6.8 and the clusterProfiler R package, and categorized into Biological Process (BP), Cellular Component (CC), and Molecular Function (MF) (Figure 12). Significance thresholds were set at *p* < 0.05 and FDR < 0.05.

In the BP category, the most enriched terms included positive regulation of apoptotic process, regulation of programmed cell death, cell cycle arrest, regulation of intracellular ROS levels, oxidative stress response, and negative regulation of cell proliferation.

In the CC category, enriched terms were primarily associated with mitochondrial and intracellular structures, including mitochondrial membrane, cytoplasm, perinuclear region, apoptosome complex, and nucleus.

In the MF category, the most significantly enriched functions included caspase activity, protein kinase binding, pro-apoptotic and anti-apoptotic protein binding, and oxidoreductase activity.

These enrichment patterns are consistent with pathways related to apoptosis, oxidative stress, and mitochondrial function, and provide supportive context for the experimental observations.

### 2.11. KEGG Pathway Analysis Reveals Enrichment of Apoptosis and ROS-Associated Signaling Pathways

**Figure 13:**
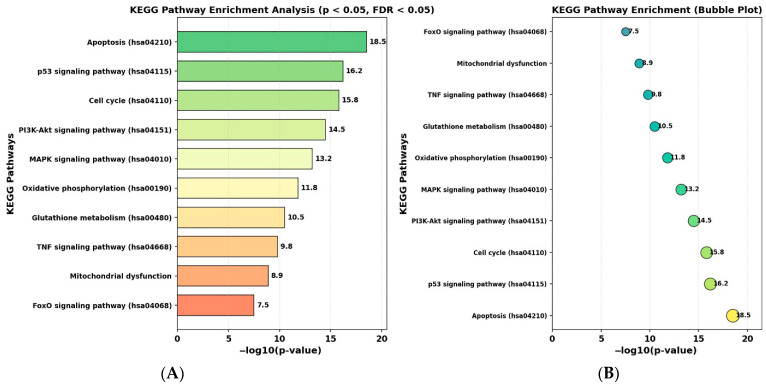
KEGG pathway enrichment analysis of RES and PAC-associated target genes. (**A**) Bar plot showing the top enriched KEGG pathways ranked by −log10(*p*-value). (**B**) Bubble plot representation of KEGG pathway enrichment, where the *x*-axis indicates −log10(*p*-value) and bubble size reflects the relative enrichment level. Significantly enriched pathways (*p* < 0.05, FDR < 0.05) include apoptosis (hsa04210), p53 signaling pathway (hsa04115), cell cycle (hsa04110), PI3K–Akt signaling pathway (hsa04151), MAPK signaling pathway (hsa04010), oxidative phosphorylation (hsa00190), glutathione metabolism (hsa00480), TNF signaling pathway (hsa04668), and pathways associated with mitochondrial function. These pathways are related to processes involving apoptosis, oxidative stress, and cell cycle regulation. These enriched pathways suggest potential associations with apoptosis-related processes, oxidative stress responses, and cell cycle regulation; however, these findings are derived from in silico analysis and should be interpreted as hypothesis-generating rather than definitive mechanistic evidence.

KEGG pathway enrichment analysis was performed using DAVID and the clusterProfiler R package (*p* < 0.05, FDR < 0.05), and the top enriched pathways are presented in Figure 13.

Among the most significantly enriched pathways were apoptosis (hsa04210), p53 signaling pathway (hsa04115), and cell cycle (hsa04110), followed by PI3K–Akt (hsa04151) and MAPK (hsa04010) signaling pathways. Additional enrichment was observed in oxidative phosphorylation (hsa00190), glutathione metabolism (hsa00480), TNF signaling (hsa04668), and pathways associated with mitochondrial function.

These enriched pathways are associated with processes related to apoptosis, oxidative stress, and cell cycle regulation. Collectively, the KEGG analysis provides a pathway-level overview that is consistent with the biological processes observed in experimental assays.

### 2.12. PPI Network Analysis Identifies Central Hub Genes Associated with Apoptosis and Cell Signaling

A protein–protein interaction (PPI) network was constructed using the STRING database (v11.5; minimum interaction score: 0.400) to explore interactions among RES and PAC-associated target genes. The network was visualized using Cytoscape v3.9.1 and analyzed with the cytoHubba plugin based on degree and betweenness centrality metrics.

**Figure 14:**
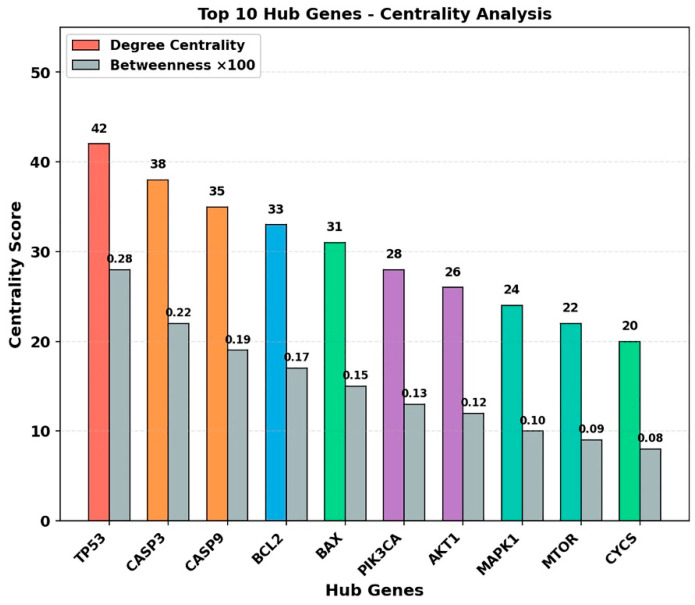
Centrality analysis of top hub genes in the PPI network associated with RES and PAC targets. Bar plot showing the top 10 hub genes identified from the PPI network using cytoHubba analysis (STRING database, minimum interaction score: 0.400). Degree centrality and betweenness centrality (×100) are presented for each gene. *TP53*, *CASP3*, *CASP9*, *BCL2*, *BAX*, *PIK3CA*, *AKT1*, *MAPK1*, *MTOR*, and *CYCS* exhibited relatively high centrality values within the interaction network, suggesting that these genes may occupy prominent positions in the predicted network topology. These genes are associated with pathways related to apoptosis, cell survival, and intracellular signaling; however, this analysis is based on computational network modeling and should be interpreted as hypothesis-generating rather than direct evidence of functional or mechanistic involvement.

The resulting network consisted of 142 nodes and 1856 edges, indicating a high level of connectivity among the target proteins. Centrality analysis identified the top 10 hub genes as *TP53*, *CASP3*, *CASP9*, *BCL2*, *BAX*, *PIK3CA*, *AKT1*, *MAPK1*, *MTOR*, and *CYCS* (Figure 14).

These hub genes exhibited relatively high degree and betweenness centrality values, suggesting that they occupy central positions within the interaction network. Notably, several of these genes are associated with apoptosis-related signaling and cell survival pathways.

Overall, the PPI network highlights a set of interconnected genes that may be involved in apoptosis, cell cycle regulation, and intracellular signaling processes, providing a systems-level context for the experimental findings.

## 3. Discussion

In the present study, the combined effects of RES and PAC were evaluated in HeLa cervical cancer cells using a multidimensional experimental framework encompassing cell viability, apoptosis, cell cycle distribution, mitochondrial membrane potential, oxidative stress, gene expression profiling, and bioinformatics analyses. The findings collectively suggest that the combination treatment is associated with enhanced cytotoxic and pro-apoptotic responses compared to single-agent conditions. These effects were accompanied by increased apoptotic cell populations, G2/M phase accumulation, mitochondrial membrane depolarization, and elevated intracellular ROS levels, together with transcriptional changes consistent with a pro-apoptotic shift. Importantly, the partial reversal of cytotoxicity and ROS accumulation by NAC indicates that oxidative stress contributes to, but does not fully account for, the observed biological effects. Taken together, these observations support the interpretation that the RES–PAC combination is associated with a multifactorial cellular response involving interconnected processes rather than a single dominant mechanism.

Both RES and PAC demonstrated dose- and time-dependent cytotoxicity, with IC_50_ values at 48 h aligning with previously reported ranges [25,26]. More importantly, the interaction analysis based on the Chou–Talalay method revealed synergistic behavior (CI < 1), suggesting that the combined treatment may enhance efficacy beyond additive effects [27]. In parallel, dose reduction indices suggest that lower concentrations of both agents may achieve comparable biological effects, which could be advantageous in reducing treatment-associated toxicity.

Selectivity is a key determinant in evaluating therapeutic potential. In this context, both RES and PAC showed higher IC_50_ values in normal HaCaT cells compared to HeLa cells, with SI values exceeding 2, indicating limited selectivity in immortalized normal cells rather than definitive tumor-specific selectivity. It should be noted that HaCaT cells are immortalized keratinocytes and do not fully represent primary normal cervical epithelial cells. Therefore, the observed selectivity should be interpreted with caution [28]. This observation is particularly relevant for translational considerations. Apoptosis analysis further supported the enhanced efficacy of the combination treatment. A marked increase in apoptotic populations was observed, affecting both early and late apoptotic fractions. While Annexin V/PI staining does not allow definitive discrimination between late apoptosis and necrosis, the overall shift toward apoptotic populations is consistent with previous findings reported for RES- and PAC-based combination strategies [29,30,31].

At the level of cell cycle regulation, PAC treatment resulted in a clear accumulation of cells in the G2/M phase, consistent with its established role as a microtubule-targeting agent. This effect is generally attributed to microtubule stabilization and disruption of mitotic spindle dynamics [32]. Notably, the combination treatment further increased G2/M phase accumulation, suggesting that RES may contribute to the observed enhancement of PAC-induced cell cycle disruption. Similar synergistic interactions between natural compounds and microtubule-targeting agents have been reported previously [33]. Mitochondrial involvement was indicated by a substantial decrease in mitochondrial membrane potential (ΔΨm) in the combination group. Although JC-1 staining reflects mitochondrial depolarization rather than direct mitochondrial outer membrane permeabilization (MOMP), the observed changes are consistent with mitochondrial dysfunction contributing to the cellular response [34].

In parallel, intracellular ROS levels were significantly elevated under combination treatment compared to single-agent conditions. As expected, H_2_O_2_ (positive control) induced a robust increase in intracellular ROS levels, confirming the sensitivity and validity of the DCFH-DA assay under the experimental conditions. This observation aligns with previous studies indicating that ROS generation has been associated with chemotherapy-induced cytotoxicity [35,36]. However, ROS measurements alone do not establish causality. To address this, NAC rescue experiments were performed. Pre-treatment with NAC reduced ROS levels and partially restored cell viability, indicating that oxidative stress contributes to the observed cytotoxicity. However, it should be noted that NAC is not a specific ROS scavenger and may exert additional effects on cellular redox balance, gene expression, and redox-sensitive signaling pathways independent of direct ROS neutralization. These findings support the interpretation that oxidative stress is contributory but not solely responsible for the observed effects. Therefore, the contribution of oxidative stress should be interpreted with caution, as the observed effects may not be exclusively attributable to ROS modulation. Importantly, the incomplete recovery suggests that ROS-independent mechanisms are also involved, which is consistent with previous reports describing multifactorial mechanisms in combination therapies [37,38,39].

At the transcriptional level, RT-qPCR analysis revealed upregulation of pro-apoptotic genes (*BAX*, *CASP9*, *CASP3*, *CYCS*) and downregulation of BCL-2, resulting in a marked shift in the *BAX/BCL-2* balance. While these changes do not directly confirm pathway activation, they are consistent with a pro-apoptotic gene expression profile reported in similar studies [40,41].

Semi-quantitative immunocytochemical observations further supported these findings. Increased caspase-9 immunoreactivity and disruption of β-tubulin organization were observed in the combination group. These results suggest concurrent effects on apoptosis-related signaling and cytoskeletal integrity, although direct mechanistic links cannot be definitively established based on these data alone. Finally, bioinformatics analyses, including GO, KEGG, and PPI network evaluations, identified enrichment of pathways associated with apoptosis, oxidative stress, and cell cycle regulation. It should be emphasized that these bioinformatics analyses are based on predicted targets obtained from databases such as SwissTargetPrediction, rather than experimentally derived gene expression datasets from the present study. Therefore, the identified pathways do not directly reflect the transcriptomic changes observed in our RT-qPCR experiments and should be considered as hypothesis-generating rather than validation of experimental findings. These analyses provide a systems-level context for the experimental observations but should be interpreted as supportive rather than confirmatory [42,43].

**Figure 15:**
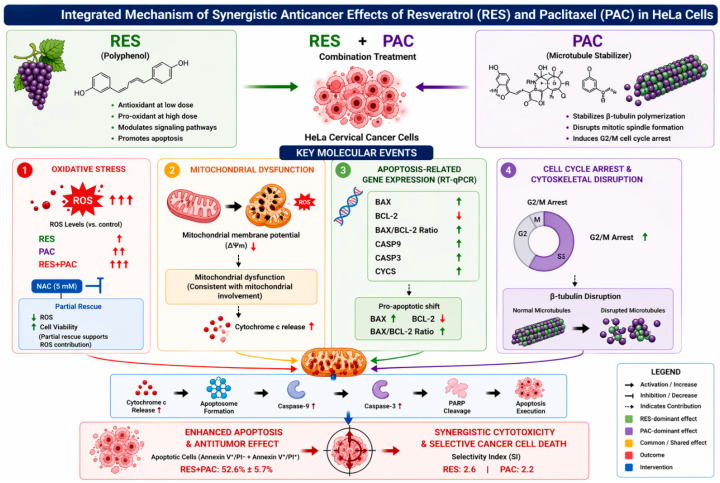
Mechanistic model of the synergistic effects of RES and PAC in HeLa cells. The schematic illustrates the integrated cellular responses induced by the combined treatment with RES and PAC, highlighting the convergence of oxidative stress, cytoskeletal disruption, and mitochondrial dysfunction. The combination markedly enhances intracellular ROS levels, which contributes to downstream signaling, as partially demonstrated by NAC mediated attenuation of both oxidative stress and cytotoxicity. In parallel, disruption of microtubule dynamics and G2 M phase arrest further amplify cellular stress. These processes collectively converge on mitochondrial dysfunction, characterized by loss of membrane potential and a shift toward a pro apoptotic gene expression profile. The coordinated engagement of these pathways results in enhanced apoptotic signaling and limited selectivity in immortalized normal cells in cancer cells. The model represents a mechanistically informed framework based on experimental observations.

The schematic model presented in Figure 15 integrates the experimental data into a unified mechanistic framework, demonstrating that the synergistic effects of RES and PAC arise from the convergence of multiple stress and signaling pathways rather than a single dominant mechanism. This integrative perspective is essential for capturing the complexity of combination therapy responses in cancer cells. The observed increase in intracellular ROS under combination treatment represents one of the contributing factors within this framework. The magnitude of ROS elevation, together with the partial reversal achieved by NAC, indicates that oxidative stress plays a critical but non-exclusive role in mediating cytotoxicity. This finding supports a model in which ROS acts as a key amplifier of downstream signaling rather than an isolated initiator.

Concurrently, PAC-induced disruption of microtubule dynamics and the resulting G2 M arrest establish a parallel axis of cellular stress. The enhanced accumulation of cells in G2 M under combination conditions suggests that RES potentiates mitotic vulnerability, thereby reinforcing the cytotoxic impact of PAC. This cytoskeletal perturbation is closely linked to mitochondrial instability, highlighting functional cross-talk between structural and metabolic stress pathways. Mitochondrial involvement emerges as a central component within the proposed model. The loss of mitochondrial membrane potential, coupled with a pronounced shift in the *BAX-BCL 2* balance and increased expression of apoptosis related genes, indicates a strong engagement of mitochondria associated signaling processes. The accompanying changes in gene expression related to cytochrome c–associated signaling and caspase-related pathways suggest activation of mitochondrial apoptotic processes, although direct evidence of cytochrome c release was not assessed and these interpretations are based on indirect indicators. It should be emphasized that MOMP and cytochrome c release were not directly measured in this study. Therefore, conclusions regarding mitochondrial mechanisms are based on indirect indicators such as ΔΨm changes and gene expression profiles.

Taken together, these findings support a model in which RES and PAC are associated with the induction of multilayered cellular stress, integrating oxidative imbalance, cytoskeletal disruption, and mitochondrial dysfunction into a coordinated pro-apoptotic response. This coordinated disruption may underlie the observed synergistic cytotoxicity and selective targeting of cancer cells. Importantly, the model should be viewed as a mechanistically informed but hypothesis-generating framework, as definitive causal relationships between these interconnected pathways remain to be fully established. Given the pleiotropic and context-dependent nature of RES, its contribution should be interpreted as modulatory rather than as a single dominant driver of the observed effects.

Several limitations of this study should be carefully considered when interpreting the findings. First, the study primarily relies on mRNA expression and immunocytochemical analyses, and does not include comprehensive protein-level validation using quantitative approaches such as Western blotting or activity-based assays. Therefore, changes in gene expression and protein presence cannot be directly interpreted as functional activation of specific signaling pathways. Second, mitochondrial involvement was inferred indirectly through measurements of mitochondrial membrane potential and apoptosis-related gene expression. Direct assessment of mitochondrial events, such as cytochrome c release kinetics, mitochondrial outer membrane integrity, or real-time bioenergetic profiling, was not performed. As a result, conclusions regarding mitochondrial mechanisms remain inferential. Third, although the NAC rescue experiment provides functional insight into the role of oxidative stress, the partial reversal observed indicates that ROS-independent mechanisms are also involved. However, alternative pathways such as autophagy, DNA damage response, ER stress, or other stress-related signaling cascades were not specifically investigated, limiting the mechanistic depth of the study. Fourth, the study was conducted in a single cervical cancer cell line HeLa, which may not fully represent the heterogeneity of cervical cancer. The use of a single cervical cancer cell line (HeLa, HPV18-positive) limits the generalizability of the findings across cervical cancer subtypes with different HPV status and genetic backgrounds. Future studies incorporating additional cervical cancer cell lines, such as HPV16-positive (e.g., SiHa) and HPV-negative models (e.g., C33A), would be important to validate the broader applicability of these findings. The absence of additional cancer cell models with different genetic backgrounds and the lack of primary tumor-derived cells limit the generalizability of the findings. Fifth, although normal HaCaT keratinocytes were included to assess selectivity, these cells do not fully recapitulate normal cervical epithelial biology. Therefore, the selectivity profile should be interpreted with caution. Sixth, the experimental design is limited to in vitro conditions and does not account for tumor microenvironment complexity, pharmacokinetics, or systemic toxicity. In vivo validation in relevant animal models is necessary to determine the translational potential of the RES and PAC combination. Finally, although bioinformatics analyses such as GO, KEGG, and PPI network construction provide a systems-level perspective, these approaches are predictive in nature and were not experimentally validated. Thus, the proposed pathways should be considered supportive rather than definitive.

Future studies should aim to validate these findings across multiple cervical cancer models with distinct genetic backgrounds and extend the analysis to in vivo systems to assess therapeutic efficacy and safety. In particular, protein-level validation of key apoptotic regulators, functional assays targeting mitochondrial dynamics, and real-time measurements of oxidative stress will be necessary to confirm the proposed mechanisms. Furthermore, the use of pathway-specific inhibitors and genetic modulation approaches may help to delineate the relative contribution of ROS-dependent and ROS-independent mechanisms. Finally, integrating these approaches with tumor microenvironment-relevant models will be critical to establish the translational potential of the RES and PAC combination.

Although these findings provide a mechanistically informed framework, they should be interpreted with caution due to the in vitro design and the use of a single cell model. Therefore, further studies incorporating protein-level validation, multiple cervical cancer models, and in vivo systems will be essential to confirm the translational relevance of the RES and PAC combination.

## 4. Materials and Methods

### 4.1. Cell Culture Conditions

Human cervical carcinoma HeLa cells (ATCC^®^ CCL-2™) and human immortalized keratinocyte HaCaT cells (Cell Lines Service, CLS, Cat. No. 300493, Eppelheim, Germany) were used in this study. HeLa cells were cultured in Dulbecco’s Modified Eagle Medium (DMEM; Gibco, Waltham, MA, USA) supplemented with 10% fetal bovine serum (FBS; Gibco) and 1% penicillin–streptomycin (Gibco). HaCaT cells were maintained under identical culture conditions using the same medium composition. Cells were incubated at 37 °C in a humidified atmosphere containing 5% CO_2_. The culture medium was renewed every 2–3 days. Cells were passaged at approximately 70–80% confluence using 0.25% trypsin–EDTA (Gibco), and all experiments were performed using cells in the exponential growth phase.

### 4.2. Chemicals and Solution Preparation

Resveratrol (RES; ≥99% purity; Sigma-Aldrich, St. Louis, MO, USA) and paclitaxel (PAC; ≥99% purity; Sigma-Aldrich) were dissolved in dimethyl sulfoxide (DMSO; Sigma-Aldrich) to prepare stock solutions at concentrations of 100 mM and 10 mM, respectively, and stored at −20 °C. Working solutions were freshly prepared by diluting the stock solutions in complete culture medium immediately before each experiment. The final DMSO concentration did not exceed 0.1% in any experimental condition. A vehicle control group containing the corresponding concentration of DMSO was included in all experiments.

### 4.3. Cell Viability Assay (Xtt)

The antiproliferative effects of RES and PAC on HeLa and HaCaT cells were evaluated using an XTT-based cell viability assay (Cell Proliferation Kit II; Roche Diagnostics, Basel, Switzerland). Cells were seeded in 96-well plates at a density of 5 × 10^3^ cells per well and allowed to adhere for 24 h. Following adhesion, cells were treated with RES (5–100 µM) and PAC (10–1000 nM) for 24 and 48 h. For combination experiments, RES and PAC were applied simultaneously under the same conditions. At the end of the incubation period, XTT reagent mixture was added according to the manufacturer’s instructions, and cells were incubated for 4 h at 37 °C. Absorbance was measured at 450 nm with a reference wavelength of 650 nm using a microplate reader (Multiskan GO, Thermo Fisher Scientific, Waltham, MA, USA). Blank wells containing culture medium without cells were used for background correction. Cell viability was expressed as a percentage relative to the untreated control group. All experiments were performed in three independent biological replicates with three technical replicates per condition. IC_50_ values were determined by nonlinear regression analysis of dose–response curves using GraphPad Prism software (version 9.0). The SI was calculated as the ratio of IC_50_ values in HaCaT cells to those in HeLa cells.

### 4.4. Combination Index Analysis

The interaction between RES and PAC was evaluated using CI analysis based on the Chou–Talalay method. RES and PAC were combined at a fixed molar ratio of 1:100, selected based on the approximate IC_50_ ranges of each agent to allow evaluation across comparable effect levels, rather than representing a strict equipotent IC_50_-based ratio. Five concentration levels (0.5×, 1×, 2×, 4×, and 8× IC_50_) were tested for each agent alone and in combination. The selected concentrations (e.g., 100 µM RES and 1000 nM PAC) correspond to near-IC_50_ to supra-IC_50_ levels derived from dose–response experiments conducted at both 24 h and 48 h time points. Cell viability data obtained from XTT assays were used to calculate the fraction affected (Fa) values. CI values were determined using CompuSyn software (version 1.0; ComboSyn Inc., Paramus, NJ, USA) according to the median-effect principle, based on dose–effect relationships of single agents and their combinations. CI values were interpreted as follows: CI < 1 indicates synergism, CI = 1 indicates an additive effect, and CI > 1 indicates antagonism. All experiments were performed in triplicate. All subsequent functional assays, including apoptosis, cell cycle analysis, mitochondrial membrane potential, and ROS measurements, were performed at 48 h using concentrations derived from 48 h IC_50_ values.

### 4.5. Apoptosis Analysis (Flow Cytometry)

The apoptotic effects of RES and PAC, alone and in combination, were evaluated by flow cytometry using Annexin V-FITC/PI double staining. Cells were treated for 48 h at IC_50_ concentrations, harvested, washed twice with cold phosphate-buffered saline (PBS), and resuspended in binding buffer according to the manufacturer’s protocol. Annexin V-FITC and PI were added, and samples were incubated for 15 min at room temperature in the dark. Single-stained controls (Annexin V-FITC only and PI only) were used for compensation to correct spectral overlap. The gating strategy included initial selection of cells based on forward and side scatter (FSC-A vs. SSC-A) to exclude debris, followed by doublet discrimination using FSC-A vs. FSC-H. Data acquisition was performed using a BD FACSCanto II flow cytometer, and at least 20,000 events were collected per sample. Data were analyzed using FlowJo software (v10.8, BD Biosciences, Franklin Lakes, NJ, USA). Cell populations were classified as follows: live cells (Annexin V^−^/PI^−^), early apoptotic cells (Annexin V^+^/PI^−^), late apoptotic cells (Annexin V^+^/PI^+^), and necrotic cells (Annexin V^−^/PI^+^).

### 4.6. Cell Cycle Analysis (Flow Cytometry)

Cell cycle distribution was analyzed by flow cytometry using PI staining. Cells were treated for 48 h at IC_50_ concentrations, harvested, washed with cold PBS, and fixed in 70% ethanol at 4 °C overnight. Following fixation, cells were washed with PBS and incubated with RNase A (100 µg/mL) at 37 °C for 30 min. Subsequently, cells were stained with PI (50 µg/mL) for 30 min at room temperature in the dark. The gating strategy included initial selection of cells based on forward and side scatter (FSC-A vs. SSC-A) to exclude debris, followed by doublet discrimination using FSC-A vs. FSC-H. Data acquisition was performed using a BD FACSCanto II flow cytometer, and at least 10,000 events were collected per sample. Cell cycle distribution (G0/G1, S, and G2/M phases) was analyzed using ModFit LT software (version 5.0, Verity Software House, Topsham, ME, USA).

### 4.7. Mitochondrial Membrane Potential

Changes in mitochondrial membrane potential were assessed by flow cytometry using JC-1 dye. HeLa cells were treated for 48 h at IC_50_ concentrations, harvested, washed with PBS, and incubated with JC-1 dye (5 µM) according to the manufacturer’s instructions for 20 min at 37 °C in the dark. After incubation, cells were washed with PBS and resuspended in fresh buffer for analysis. Data acquisition was performed using a BD FACSCanto II flow cytometer, and at least 10,000 events were collected per sample. The gating strategy included initial selection of cells based on forward and side scatter (FSC-A vs. SSC-A) to exclude debris, followed by doublet discrimination using FSC-A vs. FSC-H. Mitochondrial membrane potential was evaluated based on the shift in JC-1 fluorescence from red aggregates (FL-2 channel, ~590 nm) to green monomers (FL-1 channel, ~529 nm). The red/green fluorescence intensity ratio was calculated as an indicator of mitochondrial status, where a decrease in this ratio reflects mitochondrial depolarization.

### 4.8. Measurement of Ros

Intracellular ROS production was determined by flow cytometry using 2′,7′-dichlorodihydrofluorescein diacetate (DCFH-DA; Sigma-Aldrich). HeLa cells treated at IC_50_ doses for 48 h were washed with PBS and incubated with 10 µM DCFH-DA in serum-free DMEM at 37 °C for 30 min in the dark. Hydrogen peroxide (H_2_O_2_, 200 µM) was used as a positive control to validate the ROS detection assay. At the end of incubation, cells were washed with cold PBS, collected, and immediately analyzed by flow cytometry. A minimum of 10,000 events were acquired per sample. The gating strategy included debris exclusion (FSC-A vs. SSC-A) and doublet discrimination (FSC-A vs. FSC-H). The fluorescence intensity of DCF, formed as a result of intracellular oxidation of DCFH-DA, was measured in the FL-1 channel (excitation: 488 nm, emission: 525 nm). Background fluorescence was corrected using unstained control samples. Results were expressed as fold change in median fluorescence intensity (MFI) relative to the control group.

### 4.9. Nac Rescue Experiment

A cell viability-based rescue experiment was performed using N-acetylcysteine (NAC; Sigma-Aldrich) to evaluate the contribution of ROS to the antiproliferative effects of the RES + PAC combination. HeLa cells were seeded in 96-well plates at a density of 5 × 10^3^ cells per well and allowed to adhere for 24 h. Cells were pre-treated with 5 mM NAC for 1 h prior to drug exposure. Following pre-treatment, RES (54.3 µM), PAC (317.6 nM), or their combination was added to the respective groups, and NAC was maintained in the culture medium throughout the 48 h treatment period. Experimental groups included untreated control, NAC alone, NAC + RES, NAC + PAC, RES + PAC, and RES + PAC + NAC. Cell viability was assessed using the XTT assay at the end of the treatment period. All experiments were performed in three independent biological replicates, each with three technical replicates.

### 4.10. RT-qPCR Analysis

Quantitative real-time PCR (RT-qPCR) analysis was performed to determine the mRNA expression levels of selected genes. Total RNA was isolated using a commercially available RNA extraction kit according to the manufacturer’s instructions, and RNA concentration and purity were assessed spectrophotometrically. Complementary DNA (cDNA) was synthesized from 1 µg of total RNA using a reverse transcription kit following the manufacturer’s protocol. RT-qPCR reactions were performed using a SYBR Green master mix on a real-time PCR system under standard cycling conditions. Primer efficiency for each primer pair was validated using standard curve dilution series, yielding efficiencies between 90 and 110%. Melt curve analysis was conducted after each run to confirm the specificity of amplification. Gene expression levels were normalized to GAPDH as the reference gene and calculated using the 2^−^ΔΔCt method. Primer sequences are provided in Table 1. All experiments were performed in three independent biological replicates, each with three technical replicates.

### 4.11. β-Tubulin Immunofluorescence Staining

HeLa cells were seeded on sterile coverslips and cultured to 70–80% confluence. Cells were treated with RES, PAC, or their combination for 24 h, with untreated cells serving as control. Following treatment, cells were washed with PBS and fixed with 4% paraformaldehyde for 15 min at room temperature. Cells were then permeabilized with 0.1% Triton X-100 for 10 min and blocked with 1% bovine serum albumin (BSA) in PBS for 30 min to reduce non-specific binding. Cells were incubated overnight at 4 °C with anti-β-tubulin primary antibody (1:200 dilution), followed by incubation with Alexa Fluor 488-conjugated secondary antibody (1:500) for 1 h at room temperature in the dark. Nuclei were counterstained with DAPI for 5 min. Coverslips were mounted using antifade mounting medium, and images were acquired using a confocal laser scanning microscope under identical exposure and gain settings. β-tubulin staining was evaluated qualitatively based on microtubule organization, filament integrity, and staining intensity.

### 4.12. Immunocytochemistry

HeLa cells seeded on coverslips were treated with RES, PAC, or their combination for 48 h. Following treatment, cells were fixed with 4% paraformaldehyde and endogenous peroxidase activity was blocked using 3% H_2_O_2_. Cells were incubated overnight at 4 °C with anti-caspase-9 primary antibody (1:200 dilution), followed by incubation with HRP-conjugated secondary antibody (1:500) for 1 h at room temperature. Immunoreactivity was visualized using 3,3′-diaminobenzidine (DAB) chromogen, and nuclei were counterstained with hematoxylin. Images were captured using a light microscope under identical acquisition settings. Staining intensity was quantified using ImageJ software (version 1.54, National Institutes of Health, Bethesda, MD, USA). For each group, at least 10 randomly selected fields and a minimum of 100 cells were analyzed in a blinded manner. IOD was calculated as the sum of pixel intensities within the selected regions. In addition, H-Score was determined using the formula: H-Score = Σ (percentage of cells at each intensity level × intensity score), where intensity scores were defined as 0 (negative), 1 (weak), 2 (moderate), and 3 (strong). Data are presented as mean IOD ± standard deviation from three independent experiments.

### 4.13. Bioinformatics Analysis

Bioinformatics analyses were performed to identify potential target genes and associated signaling pathways of RES and PAC.

#### 4.13.1. Gene Ontology Analysis

Target genes associated with RES and PAC were identified using SwissTargetPrediction and TargetNet databases. Genes with a probability score > 0.5 were selected, and duplicate entries were removed. The resulting gene list was curated and converted to official gene symbols using the UniProt ID mapping tool. GO enrichment analysis was performed to evaluate biological process, cellular component, and molecular function categories. Genes without annotated GO terms were excluded from the analysis. Statistical significance for enriched GO terms was determined using a threshold of *p* < 0.05 and false discovery rate (FDR) < 0.05.

#### 4.13.2. KEGG Pathway Analysis

Kyoto Encyclopedia of Genes and Genomes (KEGG) pathway enrichment analysis was performed to identify signaling pathways associated with the selected target genes. Enrichment analysis was conducted using DAVID and clusterProfiler tools. Statistically significant pathways were defined using thresholds of *p* < 0.05 and FDR < 0.05. The most significantly enriched pathways were visualized based on enrichment scores.

#### 4.13.3. Protein–Protein Interaction Network Analysis

A PPI network was constructed to investigate interactions among proteins encoded by the selected target genes. The PPI network was generated using the STRING database (version 11.5; https://string-db.org/, accessed on 20 March 2026) with a minimum interaction score of 0.400 (medium confidence). The resulting network was visualized and further analyzed using Cytoscape software (version 3.9.1). Hub genes within the network were identified using the cytoHubba plugin based on maximal clique centrality (MCC), degree, and betweenness centrality algorithms. The top-ranked genes were selected for further analysis.

### 4.14. Statistical Analysis

All experiments were performed as three independent biological replicates (*n* = 3). Each biological replicate consisted of three technical replicates, and the mean of technical replicates was used as a single data point for statistical analysis. Data are expressed as mean ± standard deviation (SD). Normality of data distribution was assessed using the Shapiro–Wilk test. Since the data met the assumption of normality, statistical comparisons between groups were performed using one-way ANOVA, followed by Tukey’s post hoc test for multiple comparisons. A *p*-value < 0.05 was considered statistically significant. CI analysis was performed descriptively using the Chou–Talalay method, and no inferential statistical testing was applied to CI values. Statistical analyses were performed using GraphPad Prism software (version 9.0; GraphPad Software, San Diego, CA, USA).

## 5. Conclusions

In conclusion, the present study demonstrates that the combination of RES and PAC exerts a synergistic antiproliferative effect in HeLa cervical cancer cells through the coordinated modulation of oxidative stress, cytoskeletal disruption, mitochondrial dysfunction, and apoptosis-related signaling. The integration of these processes, rather than the activation of a single dominant pathway, appears to underlie the enhanced cytotoxic response observed with combination treatment.

The partial attenuation of both ROS levels and cell viability by NAC indicates that oxidative stress contributes to, but does not fully account for, the observed effects, supporting a multifactorial mechanism of action. This mechanistic interplay highlights the potential of combining natural compounds with chemotherapeutic agents to enhance efficacy while maintaining selectivity toward cancer cells.

## Figures and Tables

**Table 1:** Primer sequences used for quantitative real-time PCR.

Gene	Forward Primer (5′ → 3′)	Reverse Primer (5′ → 3′)
*BAX*	TCAGGATGCGTCCACCAAGAAG	TGTGTCCACGGCGGCAATCATC
*BCL2*	ATCGCCCTGTGGATGACTGAGT	GCCAGGAGAAATCAAACAGAGGC
*CASP3*	AGAGGGGATCGTTGTAGAAGCTG	CACAAGCGACTGGATGAACCA
*CASP9*	CCTCATCATCAACAACCTGG	AAGTCCCTTTCGCAGAAACAG
*Cyclin B1 (CCNB1)*	CCG TCC ATG CGG AAG ATC	ATG GCC AGC GGG AAG AC
*CYCS*	TTCTTCCACACCACCATGAG	GTCTGCCTTTCTCCCTTGTCT
*CDK1*	GGAAACCAGGAAGCCTAGCATC	GGATGATTCAGTGCCATTTTGCC
*ACTB (β-Actin)*	CATTGCTGACAGGATGCAGAAGG	TGCTGGAAGGTGGACAGTGAGG
*GAPDH*	GGAGCGAGATCCCTCCAAAAT	GGCTGTTGTCATACTTCTCATGG

## Data Availability

The original contributions presented in this study are included in the article. Further inquiries can be directed at the corresponding authors.

## Abbreviations

ANOVA	Analysis of variance
BAX	Bcl-2-associated X protein
BCL-2	B-cell lymphoma 2
CCNB1	Cyclin B1
CDK1	Cyclin-dependent kinase 1
CI	Combination index
CYCS	Cytochrome c, somatic
DAB	3,3′-Diaminobenzidine
DCFH-DA	2′,7′-Dichlorodihydrofluorescein diacetate
DMEM	Dulbecco’s Modified Eagle Medium
DMSO	Dimethyl sulfoxide
DRI	Dose reduction index
FBS	Fetal bovine serum
FDR	False discovery rate
FITC	Fluorescein isothiocyanate
GAPDH	Glyceraldehyde-3-phosphate dehydrogenase
GO	Gene Ontology
H_2_O_2_	Hydrogen peroxide
IC_50_	Half-maximal inhibitory concentration
IOD	Integrated optical density
KEGG	Kyoto Encyclopedia of Genes and Genomes
MAPK	Mitogen-activated protein kinase
MCC	Maximal clique centrality
MFI	Median fluorescence intensity
mRNA	Messenger RNA
MTT	3-(4,5-Dimethylthiazol-2-yl)-2,5-diphenyltetrazolium bromide
NAC	N-acetylcysteine
PAC	Paclitaxel
PBS	Phosphate-buffered saline
PI	Propidium iodide
PPI	Protein–protein interaction
RES	Resveratrol
RNA	Ribonucleic acid
ROS	Reactive oxygen species
RT-qPCR	Quantitative reverse transcription polymerase chain reaction
SD	Standard deviation
SI	Selectivity index
STRING	Search Tool for the Retrieval of Interacting Genes
XTT	2,3-Bis(2-methoxy-4-nitro-5-sulfophenyl)-2H-tetrazolium-5-carboxanilide
